# Progression of chronic liver disease to hepatocellular carcinoma: implications for surveillance and management

**DOI:** 10.1038/s44276-024-00050-0

**Published:** 2024-05-03

**Authors:** Philip J. Johnson, Anton Kalyuzhnyy, Ellen Boswell, Hidenori Toyoda

**Affiliations:** 1 Department of Molecular and Clinical Cancer Medicine, University of Liverpool, Liverpool, UK; 2 Computational Biology Facility, University of Liverpool, Liverpool, UK; 3 Department of Gastroenterology, Ogaki Municipal Hospital, Ogaki, Japan

## Abstract

**Background:**

Current opinion holds that hepatocellular carcinoma (HCC) arises as a stepwise progression from chronic liver disease (CLD) to cirrhosis and then to HCC. However, some HCCs may develop in a non-cirrhotic liver, raising uncertainty about their origin.

**Methods:**

We analysed a prospectively accrued cohort of 2592 CLD patients (median follow-up = 13 years) with no prior evidence of liver cirrhosis. To track the progression of liver fibrosis prior to HCC diagnosis, we examined serial measurements of Fib-4 (an index of liver fibrosis). We also evaluated fibrosis progression in response to antiviral treatment in patients with hepatitis C (HCV) and hepatitis B (HBV). Recognising the limitations of serologic fibrosis assessment, we correlated Fib-4 and fibrosis histology within this cohort.

**Results:**

Among HCC patients, 28% had no indication of cirrhosis prior to HCC diagnosis. Only 31% of HBV-related HCC cases followed the cirrhotic pathway. HCV patients who achieved sustained virological response (SVR) developed cirrhosis approximately 7 years before HCC diagnosis.

**Conclusions:**

Our analysis challenges the notion of cirrhosis as an obligatory stage of HCC development in CLD patients. We affirm HBV’s direct oncogenic potential and find that achieving SVR does not universally prevent HCC development. Our findings have major implications for HCC surveillance.

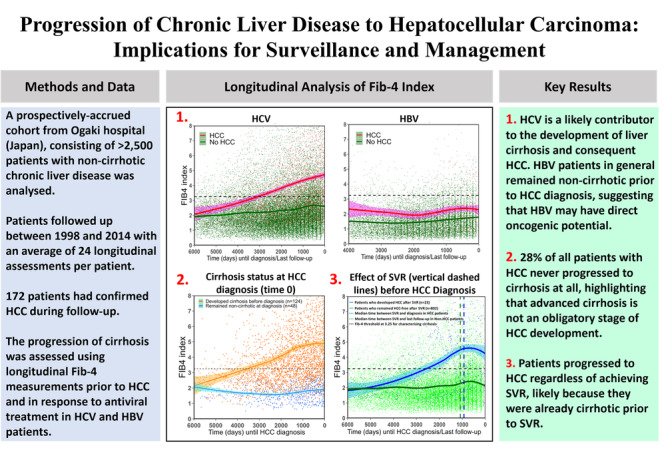

## Background

It is widely held that the majority of cases of HCC arise within a cirrhotic or severely fibrotic liver and this forms the basis of offering such patients entry into a surveillance programme with a view to establishing early diagnosis and permitting potentially curative therapy to be applied [1]. However, a small percentage of HCC cases may arise in patients without evidence of cirrhosis [2, 3]. Furthermore, the introduction of effective antiviral therapy appears to have a marked effect on the transition from CLD to cirrhosis and subsequent development of HCC.

In Japan there is a long-standing, government-funded, surveillance programme [4] and more than 70% of HCC cases are now detected by surveillance [4, 5]. Survival has increased from less than 3 months in the 1980s to more than 5 years in the most recent analyses [6]. Whilst Japanese guidelines suggest that patients with cirrhosis or advanced fibrosis are most appropriate for surveillance, the population is intensively involved within the programme and many people perceive that they may be at heightened risk of HCC because of chronic viral hepatitis [6]; such patients are not excluded from the surveillance programme even in the absence of cirrhosis. Other Asian countries such as South Korea, where chronic viral hepatitis is more prevalent than in the West, are also less restrictive in terms of patients being offered HCC surveillance [7]. As a result, and irrespective of the benefits or otherwise of this expanded surveillance approach, we had a unique opportunity to examine the progression from chronic liver disease (at a non-cirrhotic stage) to cirrhosis and/or HCC. For this study, we had access to a unique surveillance dataset from the Ogaki Municipal Hospital (Japan), which is a general hospital serving a well-defined and stable local population of approximately 400,000, where detailed serial records of patients with CLD have been kept for more than 20 years including numerous serological and clinical parameters.

Since we were interested in tracking changes in fibrosis stage over a prolonged period of follow-up, it was not possible to apply the current standard method for fibrosis assessment, namely transient elastography, as this methodology has only been recently developed. We therefore chose to rely upon the estimation of Fib-4 index, a well-established serological marker of hepatic fibrosis [8–14], to track the changes in fibrosis progression over time. However, recognising that this approach may have limitations [15], we attempted to validate Fib-4 in the current dataset by correlating Fib-4 with Metavir stages as assessed histologically where these two parameters had been measured independently within the actual dataset that we used. When combined with further data from this rigorous HCC surveillance programme that includes alpha fetoprotein (AFP) and abdominal ultrasound, we could track the progression of fibrosis and its relation to HCC development. In addition, among patients with chronic HCV infection, we were able to investigate the changes of fibrosis in response to achieving sustained virological response (SVR) [16].

## Methods

### The Ogaki cohort

We analysed serial data from a prospectively-accrued cohort of 2592 patients with CLD (Male: 50%; HBV: 28%; HCV: 51%; non-viral: 21%) without prior evidence of cirrhosis, who were recruited to a rigorous HCC surveillance programme at the Ogaki Municipal Hospital (Japan) between March 1998 and April 2014 and followed until 2021 with a median follow-up of 13.2 years. The diagnosis of CLD and the absence of cirrhosis were established at the time of enrolment into the surveillance programme by experienced clinicians using clinical, laboratory and radiological features. The diagnosis of HCC was made according to the European Association for the Study of the Liver guidelines, but the high rate of surgical resection meant that over 50% of the HCC cases were confirmed histologically. AFP levels greater than 20 ng/mL or a positive US triggered a diagnostic workup for HCC with computed tomography or magnetic resonance imaging.

Additional data was recorded for HCV patients specifically relating to their antiviral treatment, either with direct-acting antiviral (DAA) agents or interferon-based therapy (IBT), including its date and whether it led to the achievement of SVR.

We also had access to a set of 890 patients from the same surveillance programme who had confirmed liver cirrhosis at study entry, and whose data was used in this study solely to highlight the difference in HCC incidence between patients with cirrhosis at study entry and those who had non-cirrhotic CLD. As such, no further analysis was done for this group.

### Assessment of fibrosis changes over time

Serial measurements of Fib-4 index estimations were used to gain some insight into the progression of fibrosis over time in patients who were classed as non-cirrhotic at entry into the surveillance programme (*n* = 2592). Prior to the year 2000, liver biopsy was mandated before the initiation of interferon-based anti-HCV therapy and recommended thereafter. This gave us the opportunity to further validate the use of Fib-4 in estimating fibrosis changes by directly correlating Fib-4 with histologically assessed hepatic fibrosis in the actual population we are reporting on.

For classifying the development of cirrhosis over time, we used two definitions which were based on the commonly accepted Fib-4 cut-off value of 3.25 for indicating cirrhosis/severe fibrosis [10, 13, 14]. Firstly, any patients who had a Fib-4 value of >3.25 at any time during the study were considered to have developed cirrhosis during the follow-up period. Secondly, and more rigorously, any patients who had no Fib-4 values of >3.25 at any point prior to the development of HCC or at the end of the study were considered to have remained non-cirrhotic during follow-up.

To represent the changes in the degree of fibrosis over time prior to HCC diagnosis, all available serial Fib-4 measurements were smoothed using LOESS (locally estimated scatterplot smoothing) regression and presented with 95% confidence intervals. The use of LOESS was also compared against the ‘joint modelling’ approach [17].

## Results

All 2592 patients with non-cirrhotic chronic liver disease in the Ogaki cohort (Table 1) had a detailed record of serial serological features including Fib-4 (mean number of assessments per patient = 24, range 6 to 94). Within the cohort, the correlation between measured Fib-4 values and histologically assessed fibrosis showed a close alignment (Fig. S1 and Table S1), consistent with the literature [9–12, 14] on the accuracy of Fib-4 as a surrogate for the degree of fibrosis. Consequently, we tracked the changes in serial Fib-4 over time using LOESS smoothing to assess the progression of liver fibrosis within the cohort. The results were similar between using LOESS and the statistically rigorous ‘joint modelling’ approach [17] (Fig. S2).

**Table 1:** Demographics, clinical and laboratory baseline features of the cohort.

Variable	Group		
	All (*n* = 2592)	HCC^a^ (*n* = 172)	Non-HCC^a^ (*n* = 2420)
Age at baseline [years, median (range)]	58.5 (8.9–90.3)	62.7 (27.3–79.8)	57.9 (8.9–90.3)
Male, *n* (%)	1298 (50.1%)	124 (72.1%)	1174 (48.5%)
Female, *n* (%)	1294 (49.9%)	48 (27.9%)	1246 (51.5%)
Follow-up since baseline timepoint [years, median (range)]	13.2 (3.0–22.7)	13.8 (4.2–22.7)	13.1 (3.0–22.7)
Aetiology, *n* (%):			
Hepatitis C	1323 (51.0%)	131 (76.2%)	1192 (49.3%)
Hepatitis B	726 (28.0%)	29 (16.9%)	697 (28.8%)
Hepatitis B + C	5 (0.2%)	2 (1.2%)	3 (0.1%)
Other (includes 58 with confirmed NAFLD)	538 (20.8%)	10 (5.8%)	528 (21.8%)
ALBI grade, *n* (%):	*n* = 2580	*n* = 172	*n* = 2408
1	2286 (88.2%)	145 (84.3%)	2141 (88.9%)
2	289 (11.1%)	27 (15.7%)	262 (10.9%)
3	5 (0.2%)	0 (0.0%)	5 (0.2%)
ALBI score (median and range)	−2.9 (−3.8 to −1.0)	−2.8 (−3.6 to −1.3)	−3.0 (−3.8 to −1.0); *n* = 2408
Fib-4 (median and range)	1.6 (0.17–11.0)	2.4 (0.6–9.6)	1.52 (0.17–11.0)
Metavir score, *n* (%):	*n* = 2592	*n* = 172	*n* = 2420
0	1487 (57.4%)	34 (19.8%)	1453 (60.0%)
1	763 (29.4%)	80 (46.5%)	683 (28.2%)
2	316 (12.2%)	46 (26.7%)	270 (11.2%)
3–4	26 (1.0%)	12 (7.0%)	14 (0.6%)
SVR with IBT or DAA (Hepatitis C patients only)^b^:	825 (31.8%)^b^	23 (13.4%)^b^	802 (33.1%)
Nucleoside analogue (Hepatitis B patients only)^b^:	229 (8.8%)^b^	18 (10.5%)^b^	211 (8.7%)
Albumin [g/L, median (range)]	42.0 (24.0–52.0), *n* = 2580	41.0 (28.0–50.0)	43.0 (24.0–52.0), *n* = 2408
Bilirubin [µmol/L, median (range)]	10.0 (1.7–170.0), *n* = 2589	10.9 (3.3–41.7)	10.0 (1.7–170.0), *n* = 2417
Platelets [x10^3^/mm3, median (range)]	206.0 (62.0–889.0)	172.0 (71.0–470.0)	208.0 (62.0–889.0)
AFP [ng/mL, median (range)]	2.3 (0.6–12271.0)	4.9 (0.8–12271.2)	2.2 (0.6–295.8)
DCP [ng/mL, median(range)]	0.2 (0.1–484.8)	0.17 (0.1–58.4)	0.18 (0.1–484.8)
GALAD score (median and range)	−4.1 (−9.6 to 10.5)	−2.7 (−7.6 to 10.5)	−4.27 (−9.6 to 3.9)
Clinical outcome:			
Alive	2229 (86.0%)	76 (44.2%)	2153 (89.0%)
Dead	363 (14.0%)	96 (55.8%)	267 (11.0%)

All values relate to the baseline timepoint (i.e., date of first available Fib-4 measurement). Liver cirrhosis status however refers to cirrhosis status, as determined clinically, at the time of enrolment into the screening programme.

^a^HCC group includes patients who ultimately developed HCC during follow-up. Non-HCC group describes patients who remained HCC-free until their last follow-up date.

^b^HCC HCV and HBV patients were only included if they achieved SVR/received NA treatment before HCC diagnosis, respectively.

### The progression of cirrhosis in HCC patients

In total, HCC developed in 172 patients representing 7% of all cases who were non-cirrhotic at entry into the surveillance programme (Table 1 and Fig. S3). Out of those, 77 (45%) developed within the first 8 years from the study entry. The median tumour size at diagnosis was 2 cm and 122 HCC cases (71% of all HCCs) were within Milan criteria (Table 2). In total, 128 HCC patients (74%) underwent treatment with curative intent (Table 2). The HCC incidence figures translate to 5 HCC cases per 1000 patient years of follow up (PYF). The analogous figure for those with cirrhosis at entry into the surveillance programme (*n* = 890) was 31 HCC cases per 1000 PYF. The overall annual rate of progression from non-cirrhotic status to cirrhosis was 1.7% in those who did not develop HCC and 4.0% amongst those who developed HCC (Fig. S4).

**Table 2:** HCC characteristics at diagnosis. The patients were split depending on whether liver cirrhosis developed between study entry date and HCC diagnosis.

Variable	Group		
	All non-cirrhotic patients with HCC (*n* = 172)	Remained non-cirrhotic at diagnosis (*n* = 48)	Developed cirrhosis before diagnosis (*n* = 124)
Tumour characteristics			
Solitary tumours, *n* (%)	107 (62.2%), *n* = 153	35 (72.9%), *n* = 42	72 (58.1%), *n* = 111
Tumour size [cm, median (range)]	2 (1–12), *n* = 155	2 (1–12), *n* = 42	2 (1–12), *n* = 113
Vascular invasion, *n* (%)	10 (5.8%), *n* = 155	2 (4.2%), *n* = 42	8 (6.5%), *n* = 113
Within Milan criteria, *n* (%)	122 (70.9%)	32 (66.7%)	90 (72.6%)
HCC treatment, *n* (%):	*n* = 165	*n* = 47	*n* = 118
Potentially curative	128 (74.4%)	39 (81.3%)	89 (71.2%)
Palliative care	25 (14.5%)	6 (23.5%)	19 (15.3%)
Best supportive care	12 (7.0%)	2 (4.2%)	10 (8.1%)
Aetiology, *n* (%):			
Hepatitis C	131 (76.2%)	25 (52.1%)	106 (85.5%)
Hepatitis B	29 (16.9%)	20 (41.7%)	9 (7.3%)
Hepatitis B + C	2 (1.2%)	1 (2.1%)	1 (0.8%)
Other	10 (5.8%)	2 (4.2%)	8 (6.5%)

**Fig. 1:**
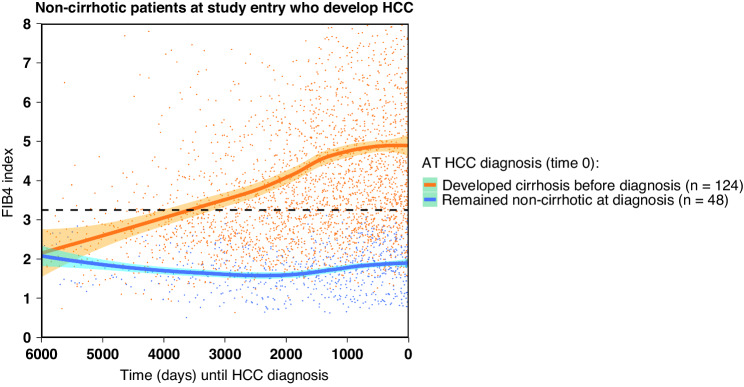
The progression of liver fibrosis prior to HCC diagnosis (time 0) in non-cirrhotic patients measured using serial changes in Fib-4 index smoothed via LOESS regression with 95% confidence intervals. Patients are grouped by whether or not they developed cirrhosis prior to HCC diagnosis (time 0). The sample size is given by *n*. The dashed horizontal black line represents a Fib-4 threshold set at 3.25 for characterising cirrhosis/severe fibrosis.

In particular, of the 172 patients developing HCC, 124 (72%) had Fib-4 values of >3.25 at some time before HCC was diagnosed indicating their eventual progression to liver cirrhosis. In those patients, cirrhosis developed, on average, around 10 years prior to HCC diagnosis (Fig. 1). The remaining 48 HCC patients (28% of all HCC patients and 2% of the overall cohort) developed HCC without ever passing through the stage of liver cirrhosis as defined by the Fib-4 threshold of >3.25 (Table 2; Fig. 1). In fact, the Fib-4 analysis presented in Fig. 1 suggests that, in the latter group, fibrosis was not progressing over time. In an attempt to validate our results using available post-resection histology data for those 48 patients, we confirmed that 35 (73%) had no evidence of cirrhosis. Our results were consistent with previous studies which found that the negative predictive value of using the 3.25 Fib-4 cut-off value for ruling out cirrhosis was over 70% [10, 14].

### The comparison of cirrhosis progression between aetiologies

We noted significant differences (*p* < 0.01, Fisher’s exact test) in aetiology between HCC patients who progressed to cirrhosis and those who remained cirrhosis-free prior to HCC diagnosis. In particular, out of the 48 HCC patients who were indicated to be non-cirrhotic prior to diagnosis based on their serial Fib-4 measurements, 20 (42%) had hepatitis B (HBV). In comparison, out of the 124 HCC patients who ultimately did progress through cirrhosis, only 9 (7%) were HBV-related. In fact, 106 (86%) of cases progressing to HCC via cirrhotic pathway were HCV-related (Table 2).

**Fig. 2:**
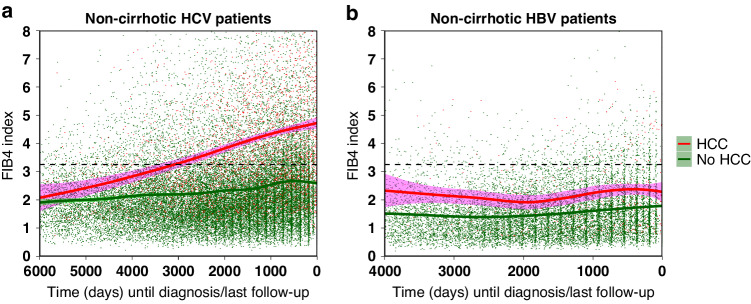
The comparison of liver fibrosis progression (measured using serial Fib-4 measurements smoothed by LOESS regression with 95% confidence intervals) between patients who developed HCC and who remained HCC-free during follow-up. The measurements are presented separately for (**a**) HCV and (**b**) HBV patients. The dashed horizontal black line represents a Fib-4 threshold set at 3.25 for characterising cirrhosis/severe fibrosis.

The relationship between HCV infection and cirrhosis progression was further highlighted by the serial Fib-4 measurements throughout the study, which revealed that in HCV-related cases cirrhosis usually progressed irrespective of HCC development (Fig. 2a). However, it is important to note that out of all 131 HCV patients with HCC who were non-cirrhotic at study entry, 25 (19%) remained non-cirrhotic at HCC diagnosis (Table 2).

Conversely, out of all 29 patients who had HBV and later developed HCC, 20 (69%) progressed directly to HCC without ever passing through a cirrhotic stage, further highlighting a direct oncogenic potential of the hepatitis B infection (Table 2). This was confirmed by the serial Fib-4 measurements for all HBV patients, where on average the cirrhotic Fib-4 threshold of >3.25 was never crossed by most HBV patients irrespective of HCC development (Fig. 2b).

Of the 58 histologically confirmed NAFLD patients who were non-cirrhotic at entry, three developed HCC and all three were cirrhotic (as assessed serologically) at the time of diagnosis. The numbers for the NAFLD group were too small for any meaningful statistical analysis.

### The impact of antiviral treatment on cirrhosis progression

**Fig. 3:**
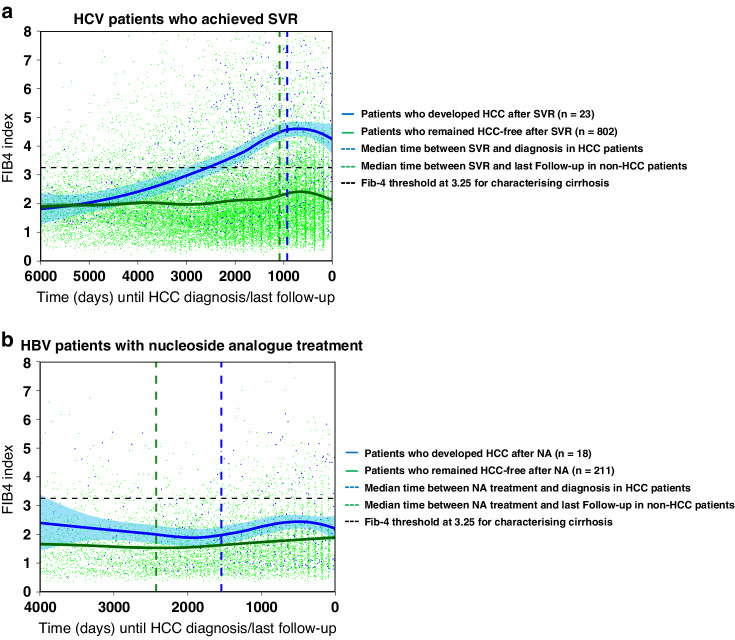
The progression of liver fibrosis (measured using serial changes in Fib-4 index smoothed via LOESS regression with 95% confidence intervals). **a** HCV patients who were diagnosed with HCC after achieving SVR and those who remained HCC-free after achieving SVR; **b** HBV patients diagnosed with HCC after undergoing nucleoside analogue (NA) antiviral treatment and those who remained HCC-free after the NA treatment. The vertical dashed lines represent the median time of achieving SVR (HCV patients) or starting the NA treatment (HBV patients) relative to the date of HCC diagnosis (time 0) or last follow-up. The sample size is given by *n*. The dashed horizontal black line represents a Fib-4 threshold set at 3.25 for characterising cirrhosis/severe fibrosis.

To assess the impact of antiviral treatment on cirrhosis progression and HCC development as measured by the Fib-4 values, we analysed the serial changes of Fib-4 in HCV patients who underwent antiviral treatment either with IBT or DAA and achieved SVR before HCC diagnosis or at some point during their follow-up if no HCC had developed (Fig. 3a). The analysis of serial Fib-4 values from patients who achieved SVR and did not develop HCC (*n* = 802) showed that on average, there was no significant change in Fib-4 values throughout the study (Fig. 3a). In comparison, in those patients who achieved SVR and later developed HCC (*n* = 23), the Fib-4 values on average increased steadily and exceeded the boundary for severe fibrosis/cirrhosis at a median time of 7 years before being diagnosed with HCC. Interestingly, we found that the median time of achieving SVR in HCC patients before diagnosis was around 2.5 years which was 4.5 years after the liver had already progressed through cirrhosis. Notably, for many of those destined to develop HCC, AFP levels had started rising around the time cirrhosis developed and prior to the achievement of SVR.

Our results also suggested that liver cirrhosis starts to progress about 14 years before HCC is detected amongst those destined to develop HCC. This observation found support in that 108 HCV patients who did not successfully achieve SVR and went on to develop HCC had a similar Fib-4 progression pattern to the 23 patients who did achieve SVR (Fig. S5), indicating that achieving SVR after the liver had progressed to cirrhosis does not prevent the development of HCC. An additional analysis of clinical and demographics features of patients who achieved SVR revealed no evidence of high alcohol consumption (Table S2).

A similar analysis was performed for the HBV patients where serial Fib-4 changes were assessed in patients who developed HCC after receiving nucleoside analogue (NA) antiviral treatment and those who remained HCC-free during follow-up (Fig. 3b). In total, out of 726 HBV patients, 229 (32%) received NA treatment. Out of those, 18 (8%) went on to develop HCC, out of whom 6 (33%) became cirrhotic before diagnosis with the remaining 12 patients (67%) never crossing the Fib-4 threshold of 3.25. Our results also showed that the NA treatment generally maintained cirrhosis at a stable level and suppressed its progression (Fig. 3b). In fact, the Fib-4 index on average remained under the 3.25 threshold in both HCC and non-HCC patients throughout their follow-up, with HCC patients having only slightly elevated Fib-4 levels (Fig. 3b).

## Discussion

The key to this research is the unique Ogaki dataset that permits us to track several clinical and serological parameters over many years before HCC is detected and even before cirrhosis develops. Unlike previous studies based on ‘cross-sectional’ data the Ogaki dataset allows us to examine the interplay between HCC and cirrhosis in unprecedented detail. The primary observation is that there is a clear pathway demonstrated between chronic liver disease and HCC that does not involve cirrhosis/severe fibrosis. As such, we propose that this is the pathway for the development of most ‘non-cirrhotic’ HCCs.

We recognise that the results of our study are dependent on the initial clinical classification of patients as non-cirrhotic and on the validity of Fib-4 as a marker for fibrosis. Nonetheless, although in current practice the presence or absence of cirrhosis would be confirmed primarily by elastography [18, 19], there is reasonable evidence in the Figure literature supporting Fib-4 as a non-invasive marker of fibrosis in liver disease, with a threshold of 3.25 being a reliable boundary between F0-2 and F3-4 Metavir stages [8–14]. Furthermore, our separate validation of Fib-4 within the current dataset offered us the opportunity to correlate Fib-4 and Metavir stages (as determined by the ‘gold-standard’ of histopathology in the same specimen) and confirm that Fib-4 is indeed a reliable indicator of the presence or absence of cirrhosis/severe fibrosis. We believe that any inaccuracy of Fib-4 is outweighed by the unique insights offered by its application.

The reported frequency with which HCC is detected in the non-cirrhotic liver varies widely from 7% to 54% but the generally accepted figure is around 20% [2, 20]. The variation is probably a result of just how the term ‘*non-cirrhotic*’ is defined. The more rigorous the definition of ‘non-cirrhotic,’ the lower the percentage is likely to be. In clinical practice, the question of whether or not cirrhosis is present in patients with chronic liver disease often arises when deciding if a patient should undergo surveillance for HCC or other complications of cirrhosis. Such decisions can be challenging. It may be difficult to establish the presence of cirrhosis consistently since, as noted in the AASLD guidelines, ‘the discrimination between severe fibrosis and compensated cirrhosis is often unclear since fibrosis can be inhomogeneously distributed within the liver’ [9]. In practice, therefore, the group undergoing surveillance is often broadened, as we have done here, to include those with ‘*severe fibrosis*’ (Metavir stage 3). By using this definition, we found that 28% of HCC cases in our study were non-cirrhotic.

Several studies suggest that non-cirrhotic HCC is more prevalent in Eastern centres such as China and Sub-Saharan Africa and one of the most striking features of our analysis is the divergence between patients with HBV and HCV in terms of their pathways to HCC. In the HBV cohort, progression to HCC generally did not appear to consistently involve cirrhosis. In contrast, most (81%) of HCV-related HCC cases did pass through cirrhosis. These findings are consistent with current opinion that HBV may lead to HCC by a direct mechanism, particularly in the setting of a regenerating liver [21, 22]. It is also consistent with international guidelines that recommend surveillance in some patients with chronic HBV even in the absence of cirrhosis [23, 24]. This observation is a further factor in the wide variation of cirrhosis in HCC patients. The difference in HCC development from 5 cases per 1000 years of patient follow up (PFU) in the non-cirrhotic patients to 31 per 1000 years PFU is striking and the disparity would be even greater if those initially non-cirrhotic patients who progressed to cirrhosis before HCC developed were included.

Nonetheless, in the study with the most rigorously defined characterisation of ‘non-cirrhotic HCC’, Chayanupatkul *et al*., found that only 10% of 8539 cases of chronic HBV in North America fell into this category [25]. This figure is much lower than that reported from Japan, Asia and Africa perhaps because of the difference in time of acquisition and hence duration of HBV exposure [26–29]. However, and of particular relevance to the current research, there may be a methodological explanation in that in most studies the classification of cirrhotic status is related to characterisation at ‘baseline’ i.e., not at the time HCC actually developed/is detected. As we show here, directly and for the first time, most non-cirrhotic CLD patients who ultimately progress to HCC do, indeed, progress to cirrhosis before they have HCC detected.

A possible limitation of this study is that it only involves patients from a single country, Japan. There is no reason to believe that Japanese HCCs present any unique characteristics and Japan is one of the few countries that has an effective surveillance programme and sufficient foresight to recognise and value long-term cohort studies. As in all areas of HCC research, the advent of effective antiviral therapy may complicate interpretation. Nonetheless, our figures stand for HCC practice internationally. Nucleotide analogues for HBV were introduced in year 2000 and interferon for HCV in 1992 before the introduction of DAAs in 2014. Development of antiviral treatment for chronic viral hepatitis is likely to accelerate the extent that prospective studies will meet even greater challenges than those experienced by the present study.

In the case of HCV, the mechanism of HCV-related HCC is perceived to be predominantly via the necroinflammatory state induced by HCV although there is evidence of a direct carcinogenic pathway [30]. Again, our results are entirely consistent with this view and the view that, once cirrhosis develops, HCC is a likely outcome even when SVR is achieved [31–33]. Our approach would be applicable to NAFLD where the pathway to, and frequency of, cirrhosis and HCC remains contentious [34–36]. However, despite the large number of cases of non-viral CLD in our cohort diagnostic criteria for NAFLD have not been sufficiently clear or consistent over the period of accrual to permit a meaningful statistical analysis.

This study illustrates the power of longitudinal datasets. In the most closely parallel study Sangiovanni et al. followed up 217 patients with compensated cirrhosis for up to 17 years. Although this detailed study provided valuable information on disease progression and its clinical consequences it started from the point of compensated cirrhosis [37]. Other longitudinal and prospective studies have employed liver stiffness to assess the degree of fibrosis as an indicator of fibrosis/cirrhosis. Such studies clearly documented that the degree of fibrosis indicates a significantly increased risk of HCC, but again most such studies only classify the degree of fibrosis at baseline and do not consider changes in the individual patient over time [38, 39]. As shown in this study a patient who is ‘non-cirrhotic on entry’ into a prospective study is not necessarily non-cirrhotic by the end of the study.

Overall, our analysis is strongly supportive currently of HCC surveillance guidelines in that the majority of HCCs arise in a cirrhotic liver, with the added proviso that patients with non-cirrhotic CLD will require monitoring to determine the point of entry into a surveillance programme. Our analyses also support guidelines in that patients with chronic HBV infection require surveillance even in the absence of cirrhosis. Notwithstanding the central role of cirrhosis in HCC development, it remains possible that cirrhosis is a ‘bystander’ acting merely as an indicator of chronicity i.e., the duration of exposure to the aetiological agent, be it alcohol or chronic viral hepatitis, and its associate necroinflammatory state, the latter being fertile ground for malignant change.

Clinical associations of HCC and chronic liver disease are generally confined to a single time point cross-sectional view. Thus, it is perceived that ‘patients without cirrhosis seldom develop HCC’ and, as such, ‘are not candidates for surveillance’. However, this study shows that even in the ‘non-cirrhotic patients’ cirrhosis may develop before or after HCC is detected. Similarly, it is perceived that most cases of HCC develop or arise in patients with ‘cirrhosis’. We can now see that it is more correct to say, most cases of HCC are detected in the cirrhotic liver. We should not make any inferences about when the cirrhosis arises. Furthermore, we cannot even be sure about the mechanistic status of cirrhosis in HCC development.

## Supplementary information

Supplementary Information

## Data Availability

The datasets generated during and/or analysed during the current study are available from the corresponding author on reasonable request.
