# Supplementary material for: Progression of chronic liver disease to hepatocellular carcinoma: implications for surveillance and management
Source: BJC Rep. 2024 May 3;2:39. doi: 10.1038/s44276-024-00050-0 (PMC11523965; doi:10.1038/s44276-024-00050-0)
Supplement: Supplementary file 1 — Supplementary Information [file 44276_2024_50_MOESM1_ESM.docx]

**Progression of Chronic Liver Disease to Hepatocellular Carcinoma: Implications for Surveillance and Management**

**Supplementary Figures and Tables**

| **Supplementary Material** | **Page Number** |
| --- | --- |
|  |  |
| **Figure S1.** Correlation between histologically assessed fibrosis stage and Fib-4 index | 2 |
|  |  |
| **Table S1.** Median Fib-4 index values with IQR in patients with HCV infection correlated with F stages | 3 |
|  |  |
| **Figure S2.** Serial Fib-4 measurements, analysed by LOESS smoothing and joint modelling with 95% confidence intervals, for patients who developed HCC and those who remained HCC-free during follow-up | 4 |
|  |  |
| **Figure S3.** Cumulative incidence of HCC cases since the start of the study in years | 5 |
|  |  |
| **Figure S4.** Cumulative incidence of cirrhosis development in patients who were non-cirrhotic at study entry | 6 |
|  |  |
| **Figure S5.** The comparison of serial Fib-4 measurements between HCV patients who underwent antiviral treatment to achieve SVR and those who failed to achieve SVR before HCC diagnosis | 7 |
|  |  |
| **Table S2.** Demographics, clinical and laboratory baseline features of patients who developed HCC despite achieving SVR and those who remained HCC-free after achieving SVR | 8 |

**Figure S1.** Correlation between histologically assessed fibrosis stage and Fib-4 index (presented as log values) in patients with chronic HCV infection prior to initiation of antiviral treatment. Horizontal red line represents Fib-4 of 3.25 which is used to diagnose cirrhosis. The patients are separated depending on whether they ultimately achieved SVR or not.

**Table S1.** Median Fib-4 index values with interquartile ranges (IQR) in patients with chronic HCV infection. The values are correlated with histologically assessed fibrosis stages F0-F4. The patients are separated depending on whether they ultimately achieved SVR or not and the number of patients for each fibrosis stage is given by *N*.

| **Fibrosis stage** | **No-SVR** | | **SVR** | | **Overall** | |
| --- | --- | --- | --- | --- | --- | --- |
|  | **N (%)** | **Fib-4 (median, IQR)** | **N (%)** | **Fib-4 (median, IQR)** | **N (%)** | **Fib-4 (median, IQR)** |
| **F0** | 51 (11.72) | 1.44 (1.04, 2.36) | 94 (15.26) | 1.28 (0.78, 1.91) | 145 (13.80) | 1.33 (0.86, 2.11) |
| **F1** | 217 (49.89) | 1.94 (1.31, 2.87) | 336 (54.55) | 1.64 (1.06, 2.55) | 553 (52.62) | 1.75 (1.16, 2.67) |
| **F2** | 104 (23.91) | 2.92 (2.00, 4.14) | 146 (23.70) | 2.51 (1.81, 3.51) | 250 (23.79) | 2.62 (1.82, 3.96) |
| **F3 + F4** | 63 (14.48) | 4.08 (2.89, 5.98) | 40 (6.49) | 3.68 (2.56, 5.22) | 103 (9.80) | 3.85 (2.63, 5.78) |


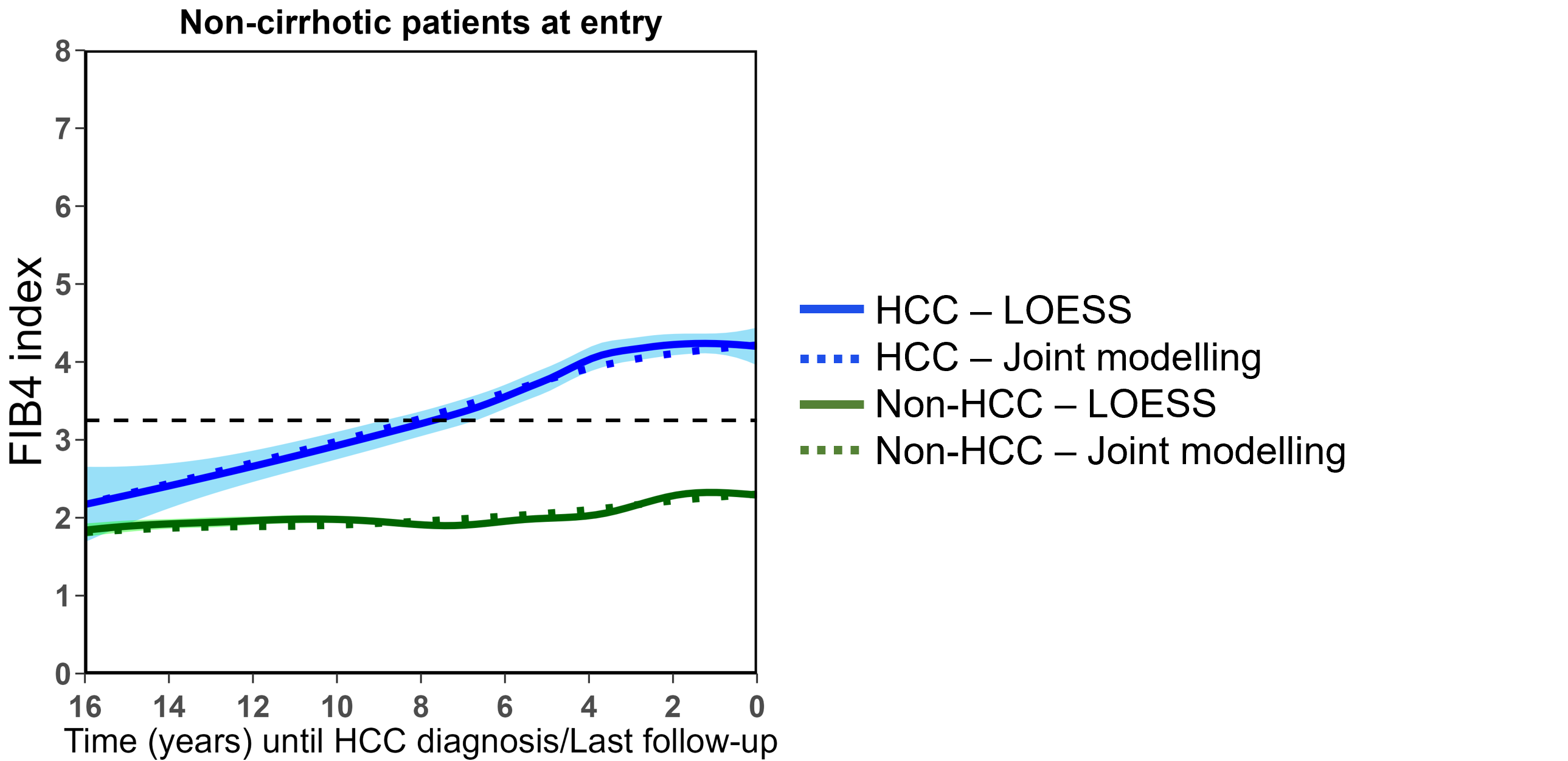


**Figure S2.** Serial Fib-4 measurements, analysed by LOESS smoothing and joint modelling with 95% confidence intervals, for patients who developed HCC and those who remained HCC-free during follow-up.


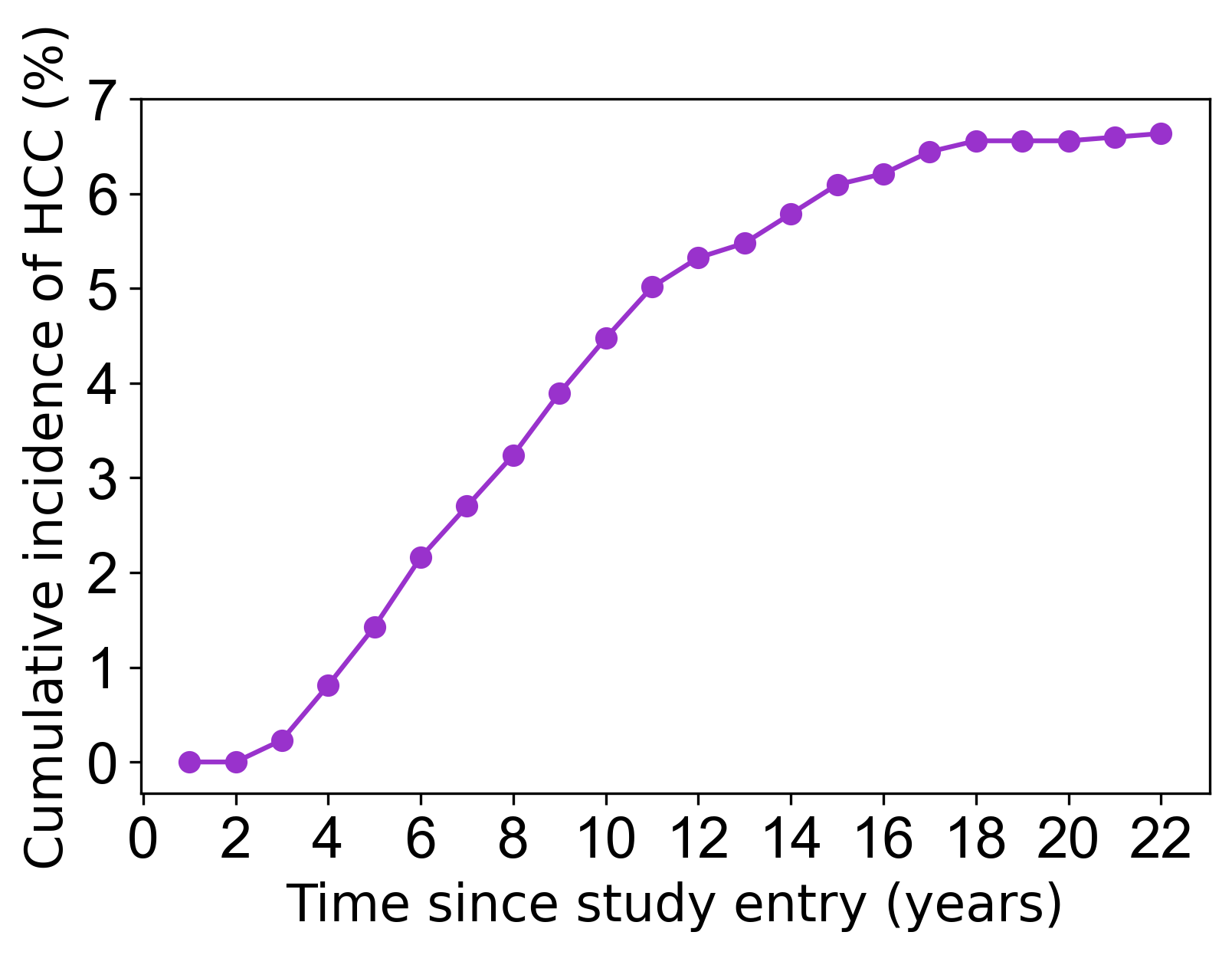


**Figure S3.** Cumulative incidence (in percentage) of HCC cases since the start of the study (time 0) in years.


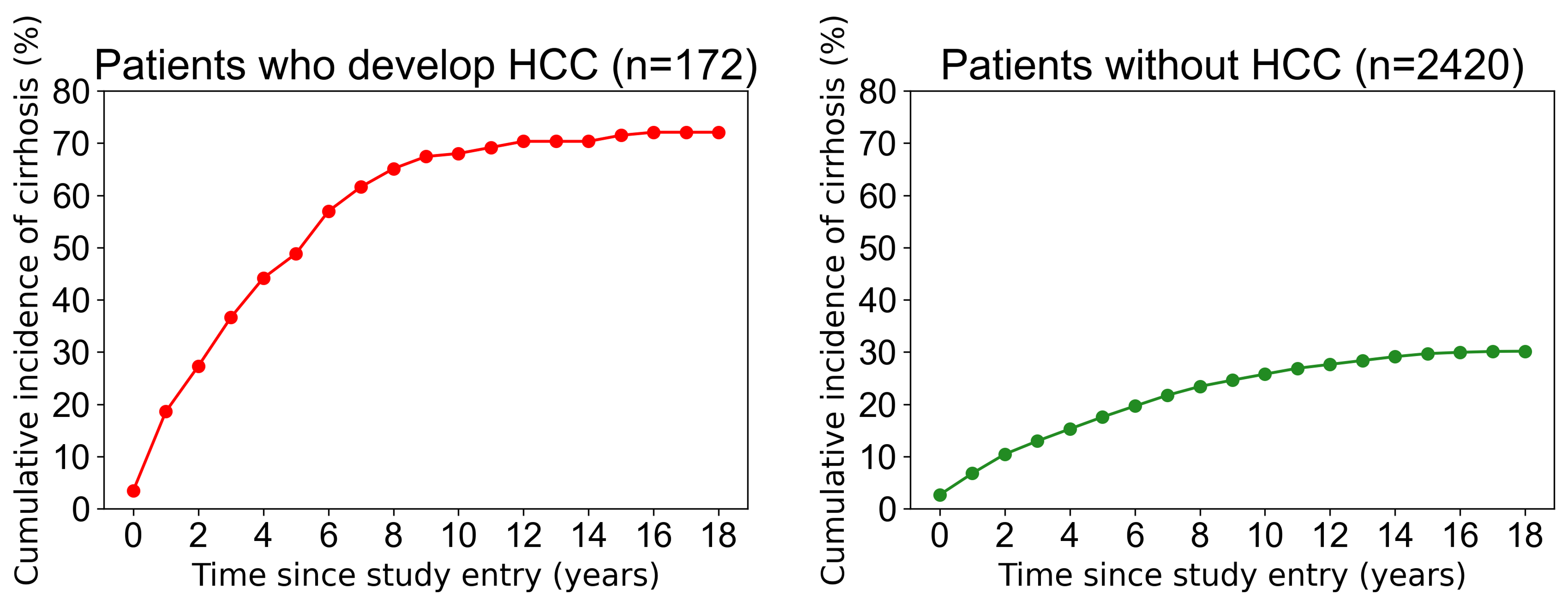


**Figure S4.** Cumulative incidence (in percentage) of cirrhosis development in patients who were non-cirrhotic at study entry (time 0) and **(A)** who were ultimately diagnosed with HCC and **(B)** who remained HCC-free during follow-up. The sample size in each group is given by *n.*


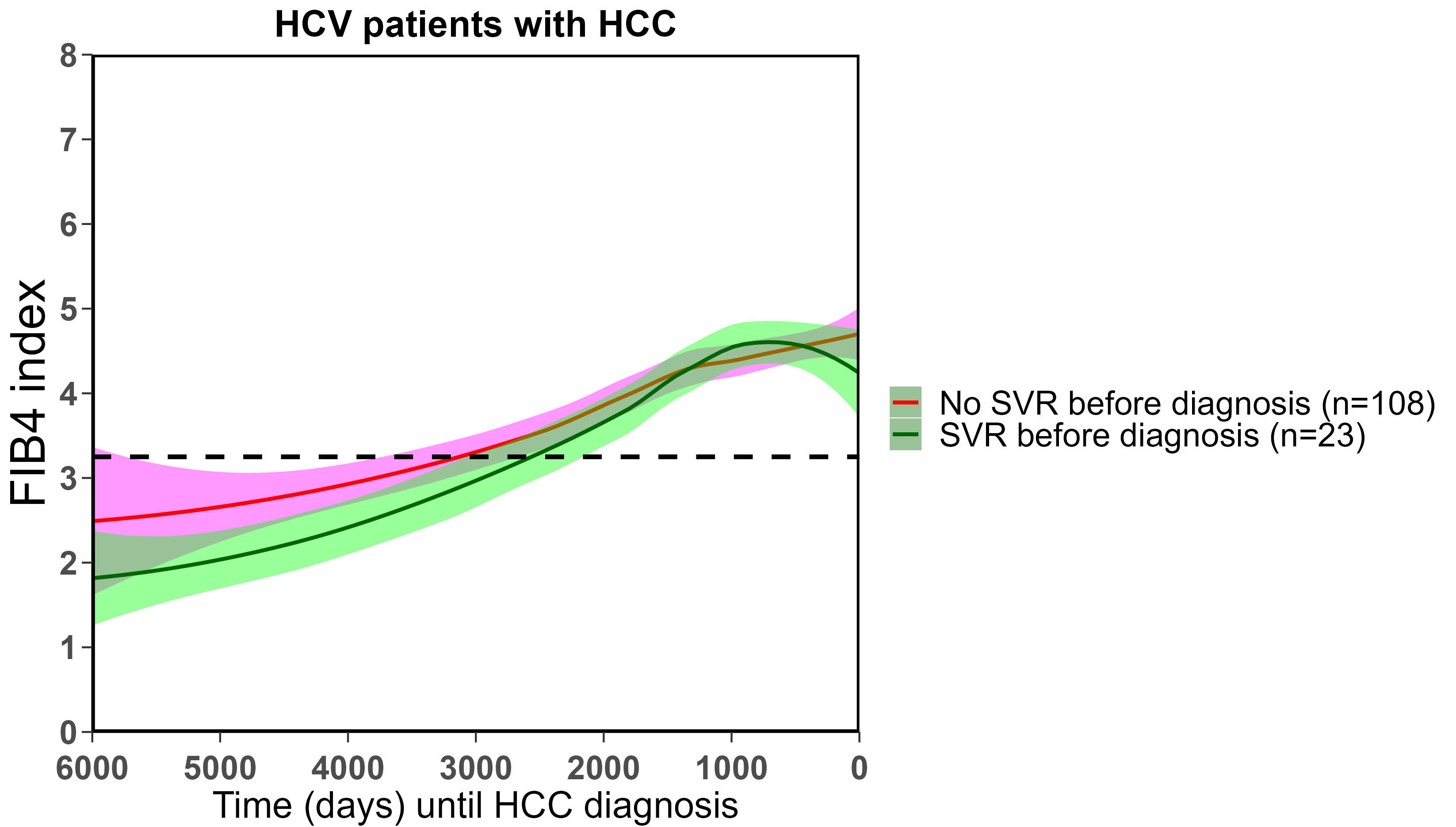


**Figure S5.** The comparison of serial Fib-4 measurements, smoothed by LOESS lines with 95% confidence intervals, between HCV patients who underwent antiviral treatment to achieve SVR and those who failed to achieve SVR before HCC diagnosis (time 0).

**Table S2.** Demographics, clinical and laboratory baseline features (i.e., recorded at the time of first available Fib-4 measurement) of patients who developed HCC despite achieving SVR and those who remained HCC-free after achieving SVR.

| **Variable** | **Group** |  |
| --- | --- | --- |
|  | **HCC after SVR (n = 23)** | **No HCC after SVR (n = 802)** |
| **Demographics, laboratory features, clinical outcome** |  |  |
| **Age at baseline [years, median (range)]** | 59.8 (51.9-73.4) | 56.8 (18.3-82.7) |
| **Male, n (%)** | 20 (87.0%) | 374 (46.6%) |
| **Female, n (%)** | 3 (13.0%) | 428 (53.4%) |
| **ALBI grade, n (%):** | n = 23 | n = 797 |
| 1 | 17 (73.9%) | 698 (87.6%) |
| 2 | 6 (26.1%) | 96 (12.0%) |
| 3 | 0 (0.0%) | 3 (0.4%) |
| **ALBI score (median and range)** | -2.8 (-3.4 to -1.9) | -2.9 (-3.8 to -1.4); n=797 |
| **FIB4 (median and range)** | 2.3 (0.7-5.2) | 1.6 (0.3-6.7) |
| **Metavir score, n (%):** | n=23 | n=802 |
| 0 | 7 (30.4%) | 475 (59.2%) |
| 1 | 11 (47.8%) | 238 (29.7%) |
| 2 | 4 (17.4%) | 86 (10.7%) |
| 3-4 | 1 (4.3%) | 3 (0.4%) |
| **Albumin [g/L, median (range)] **** | 40.0 (30.0-46.0) | 42.0 (29.0-50.0), n = 797 |
| **Bilirubin [µmol/L, median (range)]** | 10.0 (5.0-20.0) | 10.0 (3.3-101.7), n = 800 |
| **Platelets [x10^3^/mm3, median (range)]** | 185.0 (126.0-301.0) | 199.0 (82.0-561.0) |
| **AFP [ng/mL, median (range)]** | 4.7 (0.8-30.1) | 2.4 (0.8-121.8) |
| **DCP [ng/mL, median(range)]** | 0.2 (0.1-1.0) | 0.2 (0.1-251.8) |
| **GALAD score (median and range)** | -2.5 (-5.5 to -0.3) | -4.3 (-9.4 to 2.2) |
| **Alcohol consumption:** | n=19 | n=529 |
| High | 1 (5.3%) | 9 (1.7%) |
| Moderate | 4 (21.1%) | 110 (20.8%) |
| Low | 14 (73.7%) | 410 (77.5%) |
| **Clinical outcome:** |  |  |
| Alive | 15 (65.2%) | 750 (93.5%) |
| Dead | 8 (34.8%) | 52 (6.5%) |
